# In Memoriam

**Published:** 1891-10

**Authors:** 


### IN MEMORIAM.

CHICAGO, MAY 8TH, 1891.

EDITOR DENTAL REGISTER:—

At the meeting of the Chicago Dental Society, Tuersday evening, May 5th, 1891, the following resolutions on the death of Dr. William H. Atkinson, of New York, were adopted:

WHEREAS, The Chicago Dental Society having learned of the death of Dr. Wm. H. Atkinson, of New York, one of the most eminent, learned and best-known members of the dental profession, therefore, be it

*Resolved*, That in the death of Dr. Atkinson the members of this society feel a sense of personal bereavement in the loss of a much-loved and conspicuously-useful member of the profession, and while we bow in humble submission to the Divine will we desire to express our sorrow in his final exit to the unknown land beyond this world of ours. Be it further

*Resolved*, That the secretary transmit to the bereaved family of Dr. Atkinson a copy of these resolutions, and that a copy be furnished the dental journals for publication.

J. N. CROUSE,

A. W. HARLAN,

W. W. ALLPORT,

Committee.

